# Anti-HBc IgG Responses Occurring at the Early Phase of Infection Correlate Negatively with HBV Replication in a Mouse Model

**DOI:** 10.3390/v14092011

**Published:** 2022-09-11

**Authors:** Xuyang Wang, Yumeng Zhang, Yinyin Ben, Chao Qiu, Jing Wu, Wenhong Zhang, Yanmin Wan

**Affiliations:** 1 Shanghai Key Laboratory of Infectious Diseases and Biosafety Emergency Response, National Medical Center for Infectious Diseases, Department of Infectious Diseases, Huashan Hospital, Shanghai Medical College, Fudan University, Shanghai 200040, China; 2 Key Laboratory of Medical Molecular Virology (MOE/MOH), Institutes of Biomedical Sciences, Shanghai Medical College, Fudan University, Shanghai 200032, China; 3 National Clinical Research Center for Aging and Medicine, Huashan Hospital, Fudan University, Shanghai 200040, China; 4 Department of Radiology, Shanghai Public Health Clinical Center, Fudan University, Shanghai 201508, China

**Keywords:** anti-HBc IgG, AAV8-1.3HBV, HBsAg, HBV DNA, anti-HBc mAb

## Abstract

Anti-HBc IgG is usually recognized as a diagnostic marker of hepatitis B, while the functional role anti-HBc IgG in HBV infection has not been fully elucidated. In this study, we firstly investigated the relationship between the anti-HBc IgG responses and the replication of HBV using AAV8-1.3HBV infected C57BL/6N mice. Our data showed that the anti-HBc IgG responses at the early phase of infection correlated negatively with the concentrations of circulating HBsAg and HBV DNA at both the early and chronic phases of infection. This observation was confirmed by an independent experiment using AAV8-1.3HBV infected C57BL/6J mice. Furthermore, to comprehend the potential causal relationship between the anti-HBc IgG responses and HBV infection, mice were treated with an anti-HBc monoclonal antibody at three days post AAV8-1.3HBV infection. Our data showed that the anti-HBc mAb significantly suppressed the fold increase of circulating HBsAg level, and the protective effect was not affected by NK cell depletion. Collectively, our study demonstrated that anti-HBc antibodies occurring at the early phase of HBV infection may contribute to the constraint of the virus replication, which might be developed as an immunotherapy for hepatitis B.

## 1. Introduction

As one of the most frequently tested parameters for diagnosing HBV infection, anti-HBc is usually interpreted as evidence of prior infection or a risk of an ongoing HBV infection [1,2]. Isolated anti-HBc is thought to be a serological marker for occult HBV infection [3]. Although it has been studied for decades, the physiological function of anti-HBc, especially its role during HBV infection, is not completely understood.

Protective roles of anti-HBc in both acute and chronic HBV infections have been observed in early studies: Anti-HBc in the absence of HBsAg and anti-HBs may indicate recovery from an acute infection in some cases [4]. Meanwhile, it has also been suggested that persistently HBV infected individuals with no evidence of clinical disease have anti-HBc antibodies of greater affinity, compared with the chronic liver disease group [5]. These studies imply that anti-HBc antibodies might play a protective role against HBV infection. In stark contrast, more recent studies demonstrated that anti-HBc antibodies could induce hepatocytotoxicity in vitro [6] and were associated with acute liver failure caused by HBV infection [7]. Rather than a mere predictive marker, the above studies indicate that anti-HBc antibodies play a functional role in HBV infection, of which the cytotoxic effect could be a double-edged sword. On one hand, it can mediate hepatocytotoxicity; on the other hand, it may mediate elimination of HBV infected cells [8,9]. 

To clarify the controversial roles of anti-HBc antibodies, we constructed a mouse model of HBV infection by tail vein injection of a recombinant adeno-associated virus (AAV) carrying a replicable HBV genome [10]. We observed that anti-HBc IgG responses occurred at the early phase of infection were negatively associated with the replication of HBV in AAV8-1.3HBV infected mice. Through passive transfer of an HBV core antigen specific mAb, we further demonstrated that the anti-HBc IgG administered at the acute phase of AAV8-1.3HBV infection could suppress the increase of circulating HBsAg in mice.

## 2. Materials and Methods

### 2.1. Animals

Mice experiments were carried out at the Laboratory Animal Center for Drug Evaluation of School of Pharmacy (LACSP) (Fudan University, Shanghai, China). 

### 2.2. AAV/HBV Infected Mouse Model

Six- to eight-week-old C57BL/6N and C57BL/6J mice were purchased from Vital River Laboratory Animal Technology Co., Ltd. (Beijing, China). All mice were maintained under specific pathogen-free (SPF) conditions in the Laboratory Animal Center for Drug Evaluation, Fudan University. A recombinant AAV8 vector carrying 1.3 copies of the HBV genome (genotype D, subtype ayw) was purchased from PackGene Biotech (Guangzhou, China). To establish an AAV/HBV infected mouse model, the mice were infected with AAV8-1.3HBV (5 × 10^10^ GC/mouse, reconstituted in 200 μL sterile PBS) via tail vein injection. AAV8 is a liver-tropic AAV variant, and the AAV8-1.3HBV has been proved to be able to establish persistent infection in mouse liver [10,11]. In a pilot experiment, we confirmed that the AAV8-1.3HBV established persistent HBV replication mainly in mouse liver (Figure S1). 

### 2.3. Quantification of Serum HBsAg

The concentrations of HBsAg in mouse sera were measured using commercialized enzyme-linked immunosorbent assay (ELISA) kits (Shanghai Kehua Bio-Engineering Co., Ltd., Shanghai, China) according to the manufacturer’s instructions. Each sample was diluted at 1:10000 and added to the plates in duplicate. Hepatitis B virus surface antigen standard material (4 IU/mL, Beijing Kinghawk Pharmaceutical Co., Ltd., Beijing, China) was used for the quantitative determination. A secondary antibody of goat-anti-mouse IgG (HRP conjugated) (Cat# 115-035-003, Jackson Immuno Research, West Grove, PA, USA) was added to replace the original antibody in the kit and for chromogenic reaction. The absorbances at 450 nm and 630 nm were detected using a microplate reader (BioTek, Winooski, VT, USA).

### 2.4. Detections of Anti-HBc and Anti-HBs Antibodies

Antibodies to HBcAg and HBsAg were detected using commercialized ELISA kits developed by Shanghai Kehua Bio-Engineering Co., Ltd. (KHB). The detections were performed following the manufacturer’s instructions with minor modifications. Briefly, 50 μL of each diluted mouse serum (1:100) were added to the ELISA plates in duplicate and incubated for 1 h at 37 °C. Then, the plates were washed 5 times with the washing buffer. Diluted HRP-conjugated anti-mouse IgM (1:5000) (Cat# 115-035-075, Jackson Immuno Research, West Grove, PA, USA) or IgG (1:5000) (Cat# 115-035-003, Jackson Immuno Research, West Grove, PA, USA)) were used as the secondary antibody for measurements of HBV specific IgM and IgG responses, respectively. Next, the plates were washed again with the wash buffer 5 times. After washing, 25 μL of substrate A and 25 μL of substrate B (provided in the kits) were added into each well, and the plates were incubated in the dark at 37 °C for 30 min. Finally, 50 μL of the stopping buffer were added to each well. The absorbances at 450 nm and 630 nm were measured using an ELISA plate reader (BioTek, Winooski, VT, USA). 

### 2.5. Quantification of HBV DNA

HBV genomic DNA in mouse sera were detected using a quantitative PCR diagnostic kit (Sansure Biotech, Hunan, China). The detection was performed according to the manufacturer’s instructions. Briefly, 5 μL mouse sera were added to each PCR tube with 5 μL DNA lysis buffer and then mixed with the concentrated PCR reagent. PCR reactions were conducted using the Roche LightCycler 480 II (Roche Diagnostics Limited, West Sussex, UK) under the following conditions: 50 °C UNG enzyme activation for 2 min, 1 cycle; 94 °C Taq polymerase enzyme activation for 2 min, 1 cycle; 94 °C denaturation for 5 s, 57 °C annealing and extension for 30 s, 45 cycles; 25 °C cooling for 10 s, 1 cycle. The limit of detection is 100 IU/mL. 

### 2.6. Passive Antibody Transfer

5 × 10^10^ GC of AAV8-HBV1.3 was given to each mouse via tail vein injection. Three days later, 7 mice (3 female, 4 male) were injected intraperitoneally with an anti-HBc monoclonal antibody (100 μg/mouse). Eight mice (4 female, 4 male) were injected with a purified control IgG (100 μg/mouse). Another 4 mice (2 female, 2 male) were treated with the anti-HBc mAb and a NK cell depletion antibody (purified anti-Asialo-GM1 Antibody, clone# 146002, BioLegend). The anti-HBc mAb was a kind gift from Quan Yuan from Xiamen University. Peripheral blood was collected at multiple timepoints after antibody treatment. Anti-HBc IgG responses and serum HBsAg concentrations were monitored by ELISA assays.

### 2.7. Statistical Analysis

All statistical analyses in this study were done using Graphpad Prism 9.0 (GraphPad, San Diego, CA, USA). The distribution of the data was verified by the method of the Shapiro–Wilk test. Comparisons between two groups were conducted by the methods of parametric *t*-test for normally distributed data and non-parametric *t*-test for non-normally distributed data. Correlation analyses were done by Pearson correlation (for normally distributed data) or Spearman’s rank correlation (for non-normally distributed data). (*, *p* < 0.05; **, *p* < 0.01; ***, *p* < 0.001; and ****, *p* < 0.0001)

## 3. Results

### 3.1. The Recombinant AAV8-1.3HBV Established Persistent Infection in Mice

**Figure 1:**
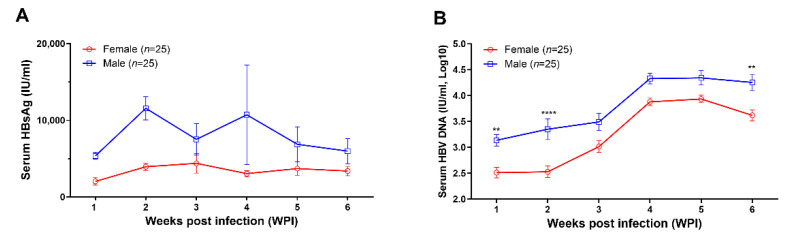
AAV8-1.3HBV established persistent infection in C57BL/6N mice. In addition, 5 × 10^10^ GC of AAV-HBV1.3 was given to each mouse via tail vein injection. (**A**) the kinetics of serum HBsAg level (IU/mL) in female (*n* = 25) and male mice (*n* = 25); (**B**) the kinetics of circulating HBV DNA levels (IU/mL, log10) in female (*n* = 25) and male (*n* = 25) mice. Data were shown as mean ± SD. Statistical analysis was performed by the method of the two-tailed *t*-test (**, *p* < 0.01; and ****, *p* < 0.0001).

After being infected intravenously with the recombinant AAV8-1.3HBV, peripheral blood was collected weekly from all mice (25 female mice and 25 male mice) for monitoring the levels of HBsAg, HBV DNA, anti-HBs IgM, anti-HBs-IgG, anti-HBc IgM and anti-HBc IgG in peripheral blood. Our results showed that persistent HBV replication was successfully established in all mice (Figure 1). The levels of both HBsAg (Figure 1A) and HBV DNA (Figure 1B) tended to higher in male mice than in female mice at all time points, while statistically significant differences were only observed for the concentrations of HBV DNA at week 1, week 2, and week 6 (Figure 1B). The HBsAg levels, especially in female mice, were relatively stable during the 6-week observation (Figure 1A). However, the circulating HBV DNA levels increased gradually after infection in both male and female mice and reached the plateau at 4 weeks post infection (Figure 1B). 

**Figure 2:**
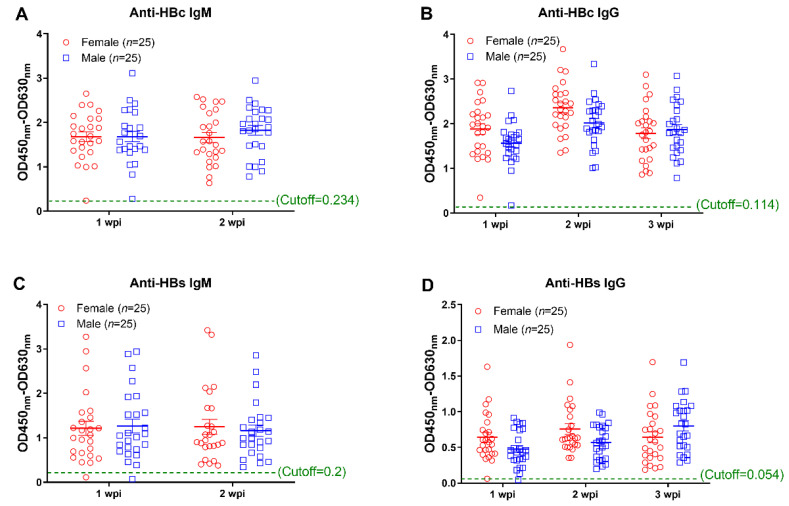
Monitoring of antibody responses against HBV core and surface proteins at the early phase infection. Antibody responses against HBc and HBs were measured by the method of ELISA. (**A**) the anti-HBc IgM responses at 1 week post infection (wpi) and 2 weeks post infection; (**B**) the anti-HBc IgG responses at 1 wpi, 2 wpi, and 3 wpi; (**C**) the anti-HBs IgM responses at 1 wpi and 2 wpi; (**D**) the anti-HBs IgG responses at 1 wpi, 2 wpi, and 3 wpi. Data were shown as mean ± SD. Statistical analysis was performed by a two-tailed *t*-test.

In addition to detecting HBV replication, we parallelly measured the HBV specific antibody responses. Our results showed that AAV8-1.3HBV infection elicited HBc and HBs specific IgM responses in most mice (except one female and one male mice) at week 1 (Figure 2A,C). At week 2, specific IgM responses were observed in all infected mice (Figure 2A,C). Similarly, we found that HBV specific IgG responses could be detected in most mice at week 1 except that one male mouse showed a negative anti-HBc IgG response, and one male and one female showed negative anti-HBs IgG responses (Figure 2B,D). At week 2 and week 3, HBc and HBs specific IgG responses were detectable in all mice (Figure 2B,D). No significant difference was observed between male and female mice at any timepoint.

### 3.2. Anti-HBc IgG Responses Are Negatively Associated with HBV Replication during the Early Phase of AAV8-1.3HBV Infection

**Figure 3:**
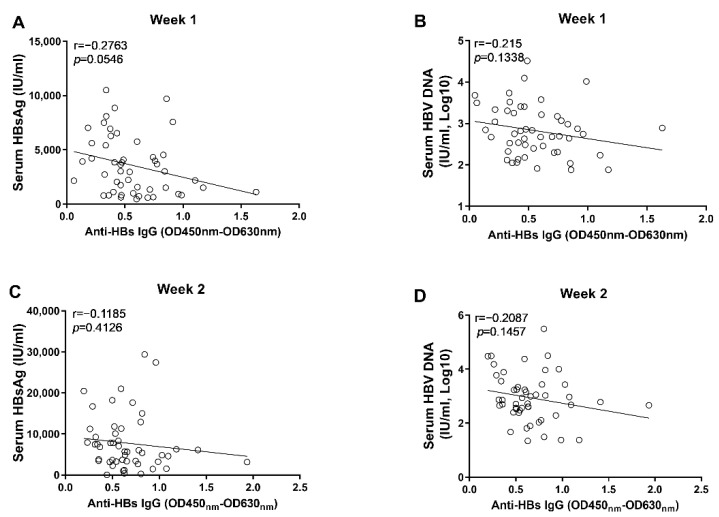
The correlation analyses between anti-HBs IgG responses and markers of HBV replication at the early phase of HBV infection. (**A**) the correlation between levels of anti-HBs IgG and concentrations of serum HBsAg at 1 wpi; (**B**) the correlation between levels of anti-HBs IgG and concentrations of serum HBV DNA at 1 wpi; (**C**) the correlation between levels of anti-HBs IgG and concentrations of serum HBsAg at 2 wpi; (**D**) the correlation between levels of anti-HBs IgG and concentrations of serum HBV DNA at 2 wpi. Statistical analysis was performed by the method of Pearson correlation (*n* = 50).

**Figure 4:**
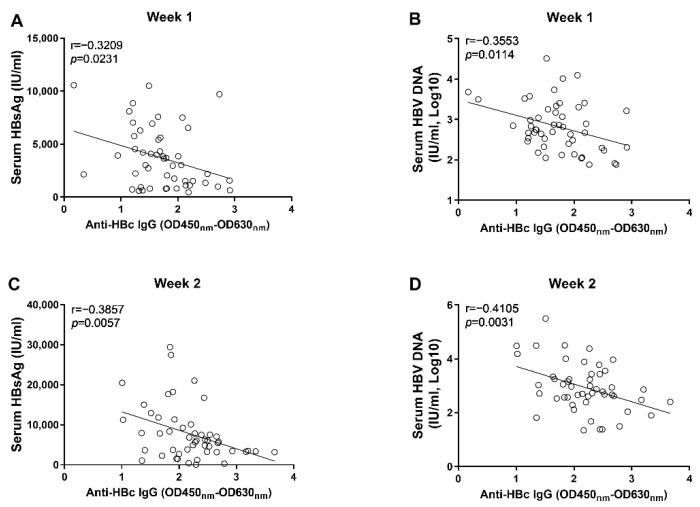
The correlation analyses between anti-HBc IgG responses and markers of HBV replication at the early phase of HBV infection; (**A**) the correlation between levels of anti-HBc IgG and concentrations of serum HBsAg at 1 wpi; (**B**) the correlation between levels of anti-HBc IgG and concentrations of serum HBV DNA at 1 wpi; (**C**) the correlation between levels of anti-HBc IgG and concentrations of serum HBsAg at 2 wpi; (**D**) the correlation between levels of anti-HBc IgG and concentrations of serum HBV DNA at 2 wpi. Statistical analysis was performed by the method of Pearson correlation (*n* = 50).

**Figure 5:**
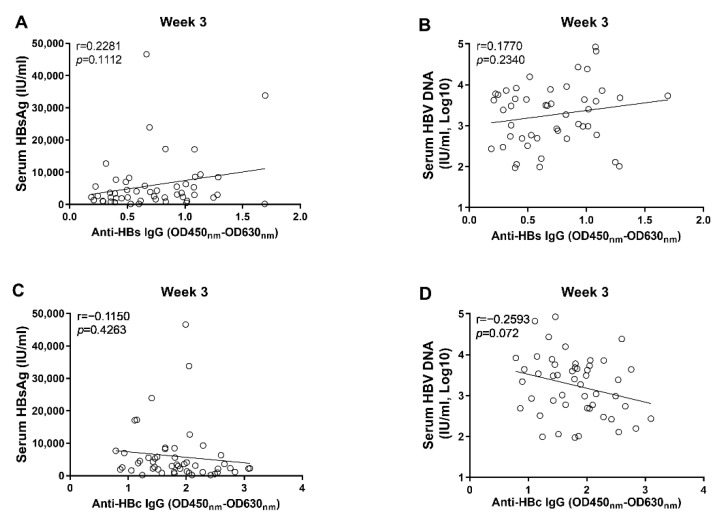
The correlation analyses between anti-HBc/anti-HBs IgG responses and markers of HBV replication at 3 wpi. (**A**) the correlation between levels of anti-HBs IgG and concentrations of serum HBsAg; (**B**) the correlation between levels of anti-HBs IgG and concentrations of serum HBV DNA; (**C**) the correlation between levels of anti-HBc IgG and concentrations of serum HBsAg; (**D**) the correlation between levels of anti-HBc IgG and concentrations of serum HBV DNA. Statistical analysis was performed by the method of Pearson correlation (*n* = 50).

Through correlation analyses, we found that the anti-HBs IgG responses at week 1 and week 2 tended to correlate negatively with levels of circulating HBsAg and HBV DNA; however, no statistical significance was observed (Figure 3). In contrast, our data showed that the anti-HBc IgG responses correlated significantly with the concurrent concentrations of circulating HBsAg and HBV DNA at week 1 and week 2, respectively (Figure 4). Moreover, although no statistically significant difference was observed at week 3, the anti-HBs IgG responses tended to correlate positively with the levels of HBsAg and HBV DNA, while the anti-HBc IgG responses tended to be negatively associated with the levels of HBsAg and HBV DNA (Figure 5). These findings collectively suggested that the anti-HBc IgG and not the anti-HBs IgG held the potential to constrain HBV replication at the early stage of infection.

### 3.3. The Early Anti-HBc IgG Responses Were Associated with the Control of HBV Replication at the Phase of Stable Infection

**Figure 6:**
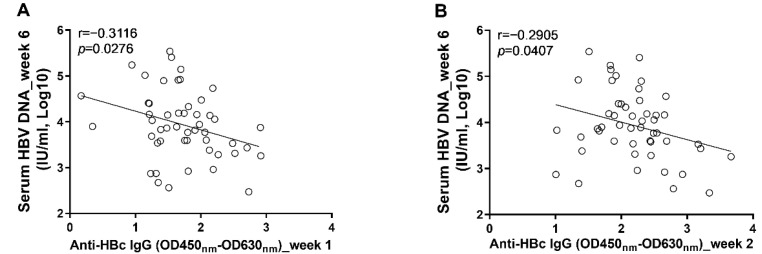
The levels of anti-HBc IgG response at the early phase of infection were associated with the levels of circulating HBV DNA at the phase of stable infection. (**A**) the correlation between levels of anti-HBc IgG at 1 wpi and concentrations of serum HBV DNA at 6 wpi; (**B**) the correlation between levels of anti-HBc IgG at 2 wpi and concentrations of serum HBV DNA at 6 wpi. Statistical analysis was performed by the method of Pearson correlation (*n* = 50).

**Figure 7:**
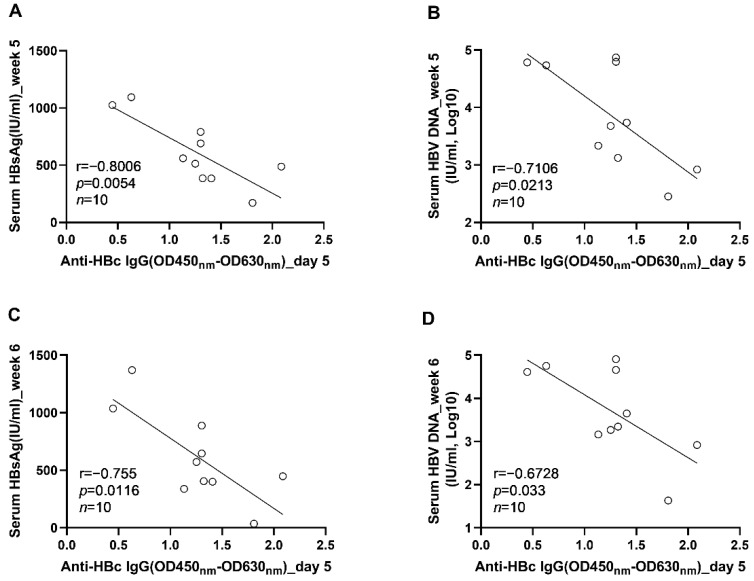
The correlations between the levels of anti-HBc IgG responses at the acute phase of infection and the levels of HBV replication at the phase of stable infection were verified in AAV8-1.3HBV infected C57BL/6J mice. (**A**) the correlation between the levels of anti-HBc IgG at 5 dpi and the serum HBsAg concentrations at 5 wpi; (**B**) the correlation between the levels of anti-HBc IgG at 5 days post infection and the serum HBV DNA concentrations at 5 wpi; (**C**) the correlation between the levels of anti-HBc IgG at 5 days post infection and the serum HBsAg concentrations at 6 wpi; (**D**) the correlation between the levels of anti-HBc IgG at 5 days post infection and the serum HBV DNA concentrations at 6 wpi. Statistical analysis was performed by the method of Pearson correlation (*n* = 10).

**Figure 8:**
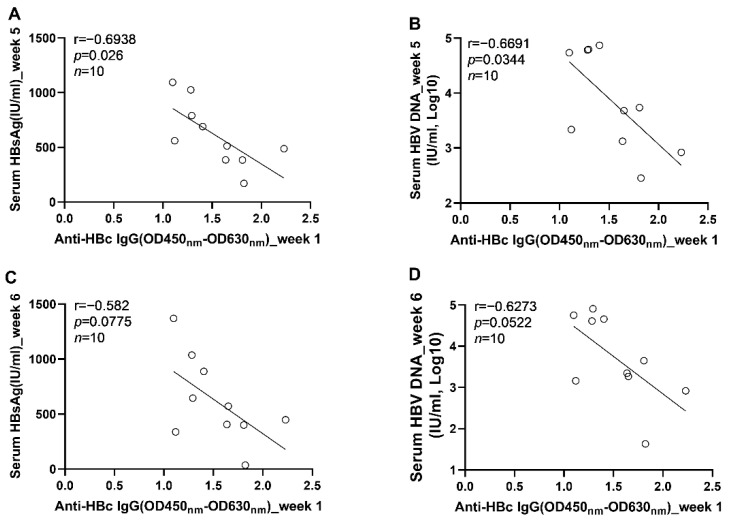
The correlation between the levels of anti-HBc IgG responses at 1 wpi and the levels of HBV replication at the phase of stable infection in C57BL/6J mice; (**A**) the correlation between the levels of anti-HBc IgG at 1 wpi and the serum HBsAg concentrations at 5 wpi; (**B**) the correlation between the levels of anti-HBc IgG at 1 wpi and the serum HBV DNA concentrations at 5 wpi; (**C**) the correlation between the levels of anti-HBc IgG at 1 wpi and the serum HBsAg concentrations at 6 wpi; (**D**) the correlation between the levels of anti-HBc IgG at 1 wpi and the serum HBV DNA concentrations at 6 wpi. Statistical analysis was performed by the method of Pearson correlation (*n* = 10).

To further clarify the role anti-HBc IgG responses in the long-term control of HBV infection, we analyzed the correlations between early anti-HBc IgG responses and markers of HBV replication at the phase of stable infection. Our results showed that the magnitudes of anti-HBc IgG responses at week 1 (Figure 6A) and week 2 (Figure 6B) correlated negatively with the concentrations of circulating HBV DNA at week 6. To verify the above findings, another 10 male C57BL/6J mice were infected with AAV8-HBV1.3 (5 × 10^10^ GC/mouse) via tail vein injection. Our data showed that the anti-HBc IgG responses detected at 5 days post infection correlated negatively with the levels of circulating HBs Ag and HBV DNA at week 5 and week 6 (Figure 7). The anti-HBc IgG responses detected at 1 wpi correlated negatively with the levels of circulating HBs Ag and HBV DNA at week 5 (Figure 8A,B). Trends of negative correlation were also observed between the anti-HBc IgG responses at week 1 and markers of HBV replication at week 6 with *p*-values close to 0.05 (Figure 8C,D).

In this study, we did not detect intrahepatic HBV markers for each infected mouse. However, to probe the effect of anti-HBc antibody on intrahepatic HBV markers, we compared percentages of HBsAg+ hepatic cells between a mouse with relatively high early anti-HBc response and a mouse with relatively low early anti-HBc responses using a method of immunohistochemistry. Our data show that the percentage of HBsAg+ hepatic cells in the mouse with high early anti-HBc is lower than that in the mouse with low early anti-HBc (Figure S2).

### 3.4. Passive Transfer of an Anti-HBc Monoclonal Antibody Early after AAV8-1.3HBV Infection Suppressed the Increase of Circulating HBsAg

**Figure 9:**
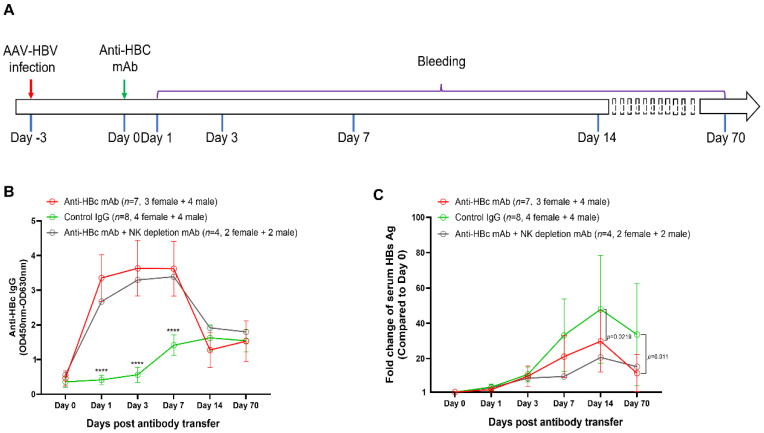
Treatment with an anti-HBc mAb suppressed the increase of serum HBsAg in AAV8-1.3HBV infected mice. (**A**) the design of the antibody transfer experiment. 5 × 10^10^ GC of AAV8-HBV1.3 was given to each mouse via tail vein injection. Three days later, seven mice (3 female, 4 male) were injected intraperitoneally with an anti-HBc monoclonal antibody (100 μg/mouse). Eight mice (4 female, 4 male) were injected with a purified control IgG (100 μg/mouse). Another four mice (2 female, 2 male) were treated with the anti-HBc mAb and a NK cell depletion antibody (purified anti-Asialo-GM1 Antibody, clone# 146002, BioLegend). As being indicated, peripheral blood was collected at day 0, day 1, day 3, day 7, day 14, and day 70. Anti-HBc IgG responses and serum HBsAg concentrations were monitored by ELISA assays; (**B**) the dynamics of anti-HBc IgG responses since the antibody treatment; (**C**) the kinetics of the fold-change of serum HBsAg since the antibody treatment. Data were shown as mean ± SD. Statistical analysis was performed by the method of a two-tailed *t*-test (****, *p* <0.0001).

To investigate the causal relationship between anti-HBc IgG and the reduction of HBV replication, mice were passively transferred with an anti-HBc mAb (*n* = 7), control mouse IgG (*n* = 8), or anti-HBc mAb plus a NK cell depletion mAb (*n* = 4) at 3 dpi (Figure 9A). Our results showed that the transfer of the anti-HBc mAb significantly increased the levels of in vivo anti-HBc IgG, which remained for at least 1 week (Figure 9B). 

Compared with the control IgG, the anti-HBc mAb significantly reduced the fold increase of circulating HBsAg level at 14 days and 70 days post antibody treatment (Figure 9C), suggesting that the anti-HBc IgG response at the early phase of infection contributed to reduce the long-term burden of HBV infection. Furthermore, our data demonstrated that the ADCC effect might not be involved in the observed protection because the administration of a NK cell depletion mAb did not negate the protective effect of the anti-HBc mAb (Figure 9C).

## 4. Discussion

While the role of antibodies in preventing virus infection is unquestionable, their role in the resolution of viral disease has not been fully classified [12]. During the past two decades, treatments of viral infections, such as CMV, HIV, EBOV, RSV, and influenza, with monoclonal antibodies have been enthusiastically pursued [13]. Although anti-HBsAg monoclonal antibodies have also been tried in treating chronic HBV infection [14], the role of humoral immunity in controlling HBV infection is relatively neglected [15]. To understand the functional role of anti-HBc in the process of HBV infection, in this study, we established an HBV infected mouse model and observed the relationship between anti-HBc/HBs antibody responses and HBV replication. 

In this study, we found that male mice tended to have higher levels of HBsAg and HBV DNA (Figure 1), which is consistent with previous clinical and experimental observations. HBV-related HCC occurs much more often in men than in women, with approximately 5–7:1 ratio [16]. In HBV transgenic mice, male mice tended to have higher viral loads than female mice [17]. Sex hormone related immune regulation is speculated to be the major underlying mechanism. However, the exact mechanism needs to be further clarified.

Our results showed that the levels of anti-HBc IgG at the 1st and 2nd week post AAV8-1.3HBV infection correlated with the concurrent levels of HBsAg and HBV DNA negatively (Figure 4). Moreover, we found that the levels of anti-HBc IgG at the 1st and 2nd week also correlated negatively with HBV DNA levels at the 6th week post infection (Figure 6). Our findings are consistent with previous observations, which show that the higher baseline anti-HBc levels are associated with better HBsAg clearance and HBeAg suppression after Peg-IFN and NA therapies [18,19,20,21]. It is noteworthy that, although the levels of anti-HBc IgG at the 3rd week post infection tended to negatively correlate with the levels of circulating HBsAg and HBV DNA, no significant difference was reached (Figure 5). This fact combined with the findings that early anti-HBc IgG responses correlated negatively with the levels of HBsAg and HBV at later time points indicate that strong anti-HBc IgG responses at the early phase of infection might be more effective in restraining HBV infection.

The protective role of HBc specific immunities has also been suggested by previous animal studies: An early study showed that immunization with hepatitis B core antigen protects chimpanzees from HBV challenge [22]. Another study showed that HBV genome with truncated HBc gene could establish more stable persistent infection in mice than the wild type HBV genome [23]. However, the causal relationship between anti-HBc IgG and HBV replication has not been clarified. To investigate this question, in this study, mice were treated with an anti-HBc monoclonal antibody at 3 days post AAV8-1.3HBV infection. Our data showed that the early anti-HBc mAb treatment significantly reduced the long-term burden of HBV infection (Figure 9). Antibody immunotherapy is widely applied in treatment of viral infections [24], but most previous efforts in developing therapeutic antibodies for HBV focus on S protein specific antibodies [25]. Our study provides evidence that anti-HBc antibodies might also contribute to the control of HBV infection, which is corroborated by previous studies showing that an HBcMAb-TAT PTD conjugate can inhibit HBV virus replication both in vitro and in vivo [26,27]. The general condition of mice was good after passive transfer of the anti-HBc antibody. However, we did not experimentally evaluate the side effects of anti-HBc antibody treatment. Moreover, the ADCC effect might not be a major mode that mediates the protection because the depletion of NK cells did not negate the protective effect of the anti-HBc mAb. Further investigations are required to clarify the underlying mechanism. 

Several limitations of this study should be noted. First, as aforementioned, the mechanism underlying the protective effect of anti-HBc IgG is not fully clarified, which requires further investigations. Second, it is unclear whether and how the specific T cell responses may contribute to the control of HBV replication in mice after AAV8-1.3HBV infection. Third, infection at week 6 might not well represent the chronic infection, a longer observation and parallel detections of liver enzyme (such as ALT and AST), HBeAg, anti-HBe antibody, and intrahepatic HBV markers will help to further characterize the impact of early anti-HBc antibody responses on chronic HBV infection. Despite these limitations, our study demonstrated that the anti-HBc IgG response occurring at the early phase of HBV infection may help to constrain the virus infection. Our findings expand previous observations that anti-Hbs mAbs are protective against HBV [28,29,30,31] and suggest that anti-HBc IgG holds the potential to be developed as an alternative immunotherapy for hepatitis B. 

## Data Availability

The datasets generated for this study are available upon request to the corresponding author.

## Supplementary Materials

The following supporting information can be downloaded at: https://www.mdpi.com/article/10.3390/v14092011/s1, Figure S1: The distribution of HBV DNA in different tissues of an AAV8-1.3HBV infected mouse. Figure S2: The detection of HBsAg^+^ cells in livers of two AAV8-1.3HBV infected mice.
