# Supplementary material for: Anti-HBc IgG Responses Occurring at the Early Phase of Infection Correlate Negatively with HBV Replication in a Mouse Model
Source: Viruses. 2022 Sep 11;14(9):2011. doi: 10.3390/v14092011 (PMC9505635; doi:10.3390/v14092011)
Supplement: Supplementary file 1 [file viruses-14-02011-s001.zip › viruses-1862903-supplementary.pdf]

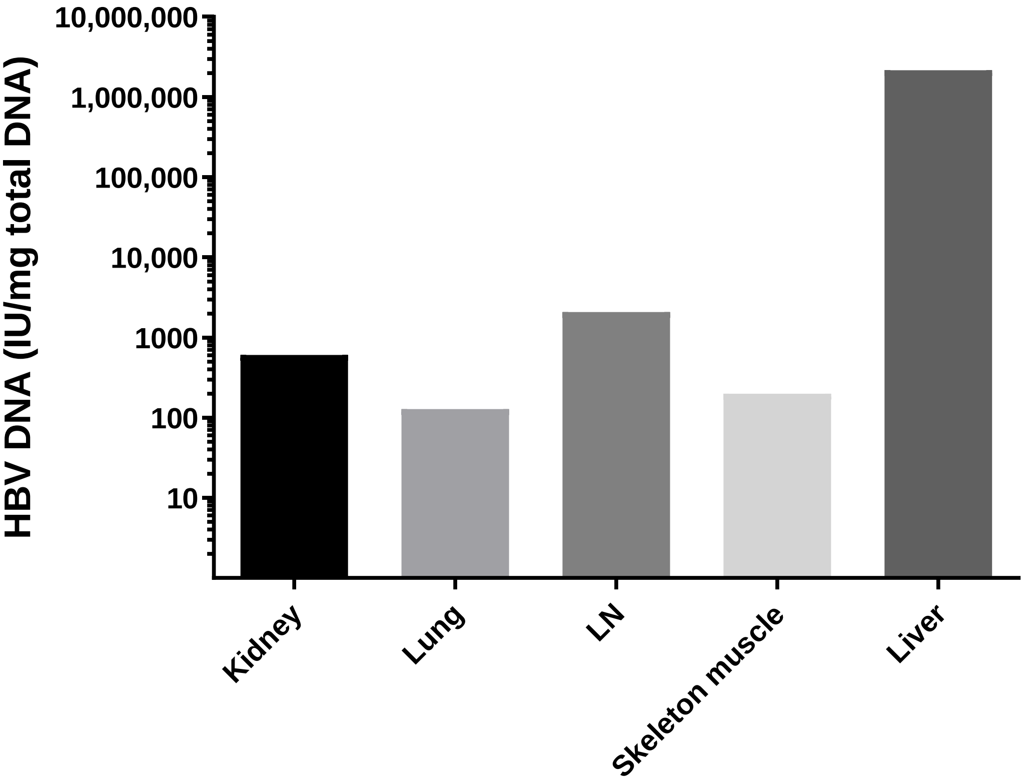

Figure S1. The distribution of HBV DNA in different tissues of an AAV8-1.3HBV infected mouse. A C57BL/6N mouse was infected with 5×10^{10} GC of AAV8-1.3HBV through tail vein injection and was euthanized at 1-week post infection. Tissue samples of kidney, lung, lymph node (LN), skeleton muscle and liver were collected. Total DNA was extracted using a Qiagen DNeasy Blood & Tissue Kit and HBV DNA was measured using a commercialized quantitative PCR kit.

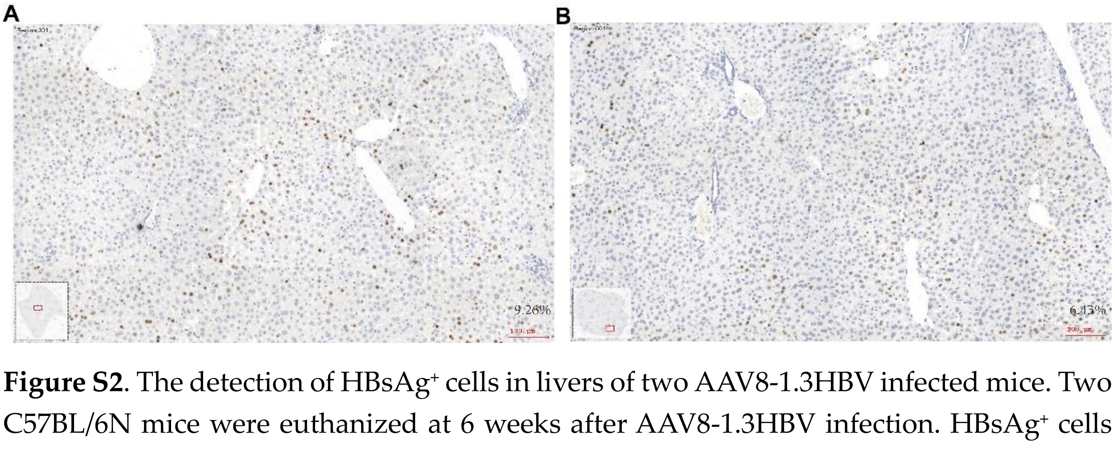

Figure S2. The detection of $HBsAg^+$ cells in livers of two AAV8-1.3HBV infected mice. Two C57BL/6N mice were euthanized at 6 weeks after AAV8-1.3HBV infection. $HBsAg^+$ cells were detected by an immunohistochemistry assay. (A) The frequency of $HBsAg^+$ cells in a mouse with relatively low early anti-HBc response (9.26%). (B) The frequency of $HBsAg^+$ cells in a mouse with relatively high early anti-HBc responses (6.43%).
